# Using an Interactive Voice Response Survey to Assess Patient Satisfaction in Ethiopia: Development and Feasibility Study

**DOI:** 10.2196/67452

**Published:** 2025-02-13

**Authors:** Dessalegn Shamebo, Anagaw Derseh Mebratie, Catherine Arsenault

**Affiliations:** 1 Department of Development Economics Ethiopian Civil Service University Addis Ababa Ethiopia; 2 Department of Health Systems Management and Health Policy School of Public Health, College of Health Sciences Addis Ababa University Addis Ababa Ethiopia; 3 Department of Global Health Milken Institute School of Public Health The George Washington University Washington, DC United States

**Keywords:** mobile phone surveys, patient satisfaction, interactive voice response, global health, surveys, Ethiopia, IVR, Africa

## Abstract

**Background:**

Patient satisfaction surveys can offer crucial information on the quality of care but are rarely conducted in low-income settings. In contrast with in-person exit interviews, phone-based interactive voice response (IVR) surveys may offer benefits including standardization, patient privacy, reduced social desirability bias, and cost and time efficiency. IVR surveys have rarely been tested in low-income settings, particularly for patient satisfaction surveys.

**Objective:**

In this study, we tested the feasibility of using an IVR system to assess patient satisfaction with primary care services in Addis Ababa, Ethiopia. We described the methodology, response rates, and survey costs and identified factors associated with survey participation, completion, and duration.

**Methods:**

Patients were recruited in person from 18 public and private health facilities in Addis Ababa. Patients’ sex, age, education, reasons for seeking care, and mobile phone numbers were collected. The survey included 15 questions that respondents answered using their phone keypad. We used a Heckman probit regression model to identify factors influencing the likelihood of IVR survey participation (picking up and answering at least 1 question) and completion (answering all survey questions) and a Weibull regression model to identify factors influencing the survey completion time.

**Results:**

A total of 3403 individuals were approached across 18 health facilities. Nearly all eligible patients approached (2985/3167, 94.3%) had a functioning mobile phone, and 89.9% (2415/2685) of those eligible agreed to be enrolled in the study. Overall, 92.6% (2236/2415) picked up the call, 65.6% (1584/2415) answered at least 1 survey question, and 42.9% (1037/2415) completed the full survey. The average survey completion time was 8.1 (SD 1.7) minutes for 15 Likert-scale questions. We found that those aged 40-49 years and those aged 50+ years were substantially less likely to participate in (odds ratio 0.63, 95% CI 0.53-0.74) and complete the IVR survey (odds ratio 0.77, 95% CI 0.65-0.90) compared to those aged 18-30 years. Higher education levels were also strongly associated with survey participation and completion. In adjusted models, those enrolled in private facilities were less likely to participate and complete the survey compared to those in public health centers. Being male, younger, speaking Amharic, using a private hospital, and being called after 8 PM were associated with a shorter survey duration. The average survey costs were US $7.90 per completed survey.

**Conclusions:**

Our findings reveal that an IVR survey is a feasible, low-cost, and rapid solution to assess patient satisfaction in an urban context in Ethiopia. However, survey implementation must be carefully planned and tailored to local challenges. Governments and health facilities should consider IVR to routinely collect patient satisfaction data to inform quality improvement strategies.

## Introduction

Collecting information on patient’s perception of health care quality is crucial to understand gaps in service provision. Patient satisfaction surveys have gained increasing attention as an essential source of information for quality improvement [1]. In high-income countries, these surveys are often paper-based and mailed to patients or delivered by email and completed on the web [2]. However, in low-income settings, these delivery methods can be problematic. Paper- and web-based surveys also assume that all recipients can read and write.

Other methods include phone surveys that have been increasingly popular, given the high rates of mobile phone ownership globally despite a relatively lower penetration rate in Sub-Saharan Africa (50%) [3]. Phone surveys with live interviewers can be effective but come with several challenges including the cost of hiring and training interviewers. They are also time-consuming for large samples and are prone to social desirability bias and to interviewer variability and bias [4,5]. Interactive voice response (IVR) surveys enable automated communications over the phone, where respondents engage with the survey using their phone’s keypad. IVR surveys offer several benefits, particularly in the context of patient satisfaction surveys. IVR surveys can be accessed by patients who may not have internet access or are not comfortable using web-based platforms. They provide a sense of anonymity, encouraging patients to provide honest feedback [6]. The automated nature of IVR ensures that every patient is asked the same set of questions in the same manner. They are also cost-effective and highly scalable and can be delivered to thousands of patients at the same time and in multiple languages, leading to very short data collection periods for real-time feedback [7-9]. Furthermore, an IVR system can make multiple calls both during and outside of normal business hours, allowing respondents to participate in the survey at their convenience [10]. Given these benefits, they have the potential to be integrated into the routine operations of health systems.

Nonetheless, IVR surveys can face a unique set of limitations and challenges. First, they may not be suitable for individuals who are not comfortable with automated phone systems. They may lead to lower engagement, greater response fatigue, a higher rate of survey abandonment, and thus a lower overall response rate compared to other survey delivery methods. People who are not technology-savvy may struggle with navigating IVR systems. Despite these limitations, IVR surveys have been used for various purposes such as for patient satisfaction assessment after endoscopy [11], evaluation of child and adolescent psychiatric outpatient treatment [8], and decision-making about sun protection [12]. Yet, there is little published evidence on the acceptability and feasibility of using IVR to collect patient feedback in Ethiopia. We found only 2 peer-reviewed studies that discussed the feasibility of IVR in Ethiopia [13,14]. These studies assessed the feasibility of IVR messages for targeted client communication. One working paper assessed the feasibility of an IVR survey in Ethiopia to obtain nationally representative estimates at population levels [15]. To our knowledge, there has been no study assessing the use of IVR to measure patient satisfaction in Ethiopia.

In this paper, we assessed the feasibility of using an IVR survey to assess patient-perceived quality of primary care services in 18 health facilities in Addis Ababa, Ethiopia. We described the methodology, acceptability, cost, response rate, completion rate, and the time taken to complete the full survey. We also assessed the factors associated with survey participation, completion, and duration. These findings may be helpful for others conducting studies among patients or considering using IVR in a similar setting.

## Methods

### Study Design and Recruitment

This study was conducted in Addis Ababa, Ethiopia, in June to July 2024. Phone numbers and consent from primary care patients were obtained in person at health facilities. The study aimed to compare patient-perceived quality of primary care services across 4 types of facilities: public health centers, public general hospitals, private clinics, and private hospitals. Four facilities from each category were randomly selected from health management information system lists with the aim of enrolling a minimum of 100 patients per facility.

Enrollment was limited to patients receiving 11 common primary care services, including for chronic diseases, injuries, infectious diseases, gastrointestinal conditions, respiratory diseases, prenatal care, and family planning. Eligible participants were those who understood Amharic or Afaan Oromo, were aged 18 years and older, were at the facility for their own care, had received one of the selected services as an outpatient, and had a functioning mobile phone. Hospitalized individuals were excluded. Data collectors identified eligibility, obtained consent, and collected sex, educational level, mobile phone number, and preferred contact time for the IVR survey. No other personal identifiers were collected.

The recruitment period lasted 14 days. Each day, phone numbers were uploaded to the IVR platform. One day after enrollment, participants received an SMS text message at their preferred time to remind them of the study, followed by a call within 10 minutes to complete the IVR survey. During recruitment, respondents listened to a sample IVR question to familiarize themselves with the system. They were advised to respond honestly and to find a quiet location for the survey.

The IVR survey was programmed using the EngageSpark platform. The survey questions and response options were recorded in a recording studio in Amharic and Afaan Oromo and uploaded to the IVR platform. The IVR survey recordings were narrated by a female, given evidence that IVR surveys narrated by female individuals lead to higher response rates and can prevent the incidence of domestic violence [16]. The first call for the IVR survey took place 1 day after the primary care visit. If the participant did not pick up, the system automatically called the respondent back after 30 minutes, 1 hour, 90 minutes, 1 day, 1 day and 1 hour, 2 days, and 2 days and 1 hour. For respondents who completed the full survey, the EngageSpark platform automatically sent the airtime top-up to the respondent’s phone.

The patient satisfaction survey included 15 questions covering structural quality (eg, perceived quality and availability of equipment, medicine, and diagnostic test), competence of health providers, respectful care, patient-centered care, and user experience. The survey instrument is provided in Multimedia Appendix 1. Participants were also asked to report their monthly household income and how likely they were to recommend the health facility to a family member or friend. Most questions used a 5-point Likert scale. Among 15 questions asked, 13 items on the quality and cost of care were rated on a 5-point Likert scale (from 1=very poor to 5=very good). The question on endorsement of the health facility was answered on a 4-point Likert scale (1=not at all likely to 4=very likely). The household income question was categorized into 6 income groups.

Respondents were instructed to use their mobile phone touchpad to answer questions. Respondents were allowed to repeat or skip a question. A message was recorded to inform participants if they had pressed an invalid key. The survey question was repeated if respondents pressed an invalid key or did not answer for more than 20 seconds. The call was disconnected if they pressed an invalid key 3 times or failed to respond after a question was repeated twice.

### Measures

The outcomes of interest in this study were participation in the IVR survey, completion of all survey questions, and time taken to complete all 15 survey questions (among those who completed the survey). Participation in the IVR survey was measured using a dummy variable, which was coded as 1 if the individual picked up the survey call and answered at least 1 of the survey questions and 0 otherwise. Similarly, the completion outcome was constructed as a dichotomous variable, coded as 1 if the respondent completed all 15 questions and 0 if they did not. Duration was based on the time taken in minutes to answer all 15 survey questions among those who completed the full survey. To assess the correlates of survey participation, completion, and duration, our analysis included a series of covariates collected during the in-person recruitment. These were the respondent’s sex, age, educational achievement, type of primary health care service received, type of health facility used, preferred language (Amharic or Afaan Oromo), and preferred survey time.

### Statistical Analysis

We used descriptive statistics to describe patient demographics, enrollment, survey response, completion rates, and survey duration. A Heckman probit regression model was used to identify factors influencing survey participation (answering at least 1 question) and completion (answering all questions), with the first step determining participation factors (selection model). We chose the Heckman probit model over the standard probit model due to its advantages in predicting outcomes when there is a dependency between the selection model and the outcome model as well as a correlation of error terms between the 2 models [17,18]. The participation model specification was:

S*=*ZB*+*ε*

Where S* is a latent variable representing the participation decision in the IVR survey, *Z* is a vector of covariates that include the respondents’ characteristics (sex, age, education level, timing of the IVR survey, type of facility visited, type of care received, and the patient’s preferred language), *B* represents the coefficients of interest, and *ε* is the error term. The second step of the model aimed to identify the factors associated with the completion of the IVR survey (outcome variable), conditional on participation in the survey. This equation used the inverse Mills ratio, derived from the participation equation, to overcome selection bias.

The outcome (completion) model was specified as:

Y*=*Xψ*+*λ*+*u*

Where Y* is a latent variable representing the outcome, *X* is a vector of covariates that includes all factors listed in the selection model plus number of reconnections made to complete the survey, *ψ* represents the coefficients to be estimated, *λ* is the inverse Mills ratio from the participation equation, and *u* is the normally distributed error term. Adding *λ* adjusts the outcome equation for more accurate estimates of the relationship between participation and covariates. Language was used as a selection variable for participation in the IVR survey, with Afaan Oromo speakers from rural areas around Addis Ababa being more likely to participate than those from urban areas.

For those who completed the full survey, a Weibull regression model was used to identify the factors associated with the time taken to complete the survey. The main cost driver of an IVR survey is the airtime cost while a respondent answers the survey. Identifying factors influencing survey duration is helpful due to cost implications. Duration data are often skewed, with many early events and few late ones. The Weibull model is versatile for providing insights about the effects of covariates on the timing of events (survey completion).

### Ethical Considerations

The study was approved by the Addis Ababa University College of Health Sciences institutional review board (approval 057/23/SPH). Ethics approval was also obtained from the Addis Ababa City Health Bureau. Only those participants who provided verbal consent were interviewed. All data were deidentified to ensure participant privacy and confidentiality. Participants who completed the full survey received approximately US $0.86 mobile phone airtime top-up.

## Results

### Recruitment

Table 1 reports the number of individuals who were approached and enrolled in the study. Of 3403 individuals approached at the study health facilities, 3167 (93.1%) were receiving outpatient care for themselves, and 2985 (87.7%) had a functioning mobile phone with them at the time. The majority (n=2685, 78.9%) had received 1 of 11 eligible primary care services. Of these, 2415 (86.9%) agreed to participate, provided a valid phone number, and were enrolled in the IVR survey.

**Table 1:** Recruitment results: eligibility and consent to participate in the interactive voice response survey.

		Values, n (%)
**Number of people approached (n=** **3403)**		
	Receiving care for themselves	3238 (95.2)
	Did not spend the night at the facility	3167 (93.1)
	Had a functioning mobile phone	2985 (87.7)
	Received eligible primary care services^a^	2685 (78.9)
**Eligible to participate in the study (n=** **2685** **)**		
	Agreed to participate	2604 (97)
	Agreed to provide their mobile phone number	2469 (92)
	Provided a valid mobile phone number	2415 (89.9)

^a^Participants were eligible to participate in the study if they received at least 1 of the following services: care for hypertension or high blood pressure, care for diabetes or high blood sugar, care for kidney disease, care for an injury, prenatal care, family planning services, care for tuberculosis, HIV or AIDS or a sexually transmitted infection (including testing or treatment), care for diarrhea or gastrointestinal conditions, or care for a respiratory illness.

### IVR Survey Completion and Duration

Of 2415 individuals enrolled in the IVR survey, 2236 (92.6%) participants picked up the call on their mobile phone a day later. However, 16.6% (n=400) of these respondents only listened to the introduction, and another 10.4% (n=252) listened to the first question but failed to answer it, leading to 1584 (65.6%) respondents answering at least 1 question. Among them, 8.6% (n=208) answered part of the survey and hung up, and 14% (n=339) answered part of the survey but were disconnected by the system (for failing to respond or pressing invalid keys more than twice). Finally, 1037 (42.9%) respondents completed the full survey (answered all 15 questions). Those who completed the survey took on average 8.5 (SD 1.68) minutes to answer all questions, with a median of 7.55 (IQR 6.54-8.58) minutes, a minimum of 4 minutes and 4 seconds, and a maximum of 14 minutes and 59 seconds (Table 2).

**Table 2:** Interactive voice response (IVR) survey response rate.

			Values, n (%)
**Enrolled in the IVR survey (n=2415)**			
	Picked up the IVR survey call	2236 (92.6)	
	Answered at least 1 question	1584 (65.6)	
**Participated in the IVR survey (n=1584)**			
	Answered at least 1 question but the system ended the call^a^	339 (14)	
	Answered at least 1 question but hung up without completing the survey	208 (8.6)	
	Answered the full survey	1037 (42.9)	

^a^The IVR system disconnected the call if the user did any of the following 3 times: failed to reply within 20 seconds or pressed an invalid key.

### Characteristics of the Study Respondents

Among the 2415 enrolled individuals, 1584 (65.7%) participated in the survey by responding to at least 1 survey question. The characteristics of the eligible individuals by participation status are reported in Table 3. A little over half of the individuals were female (n=1398, 57.9%), and the majority were younger than 40 years of age. Educational attainment varied, with nearly half of the respondents having education above the secondary level, and only 5.6% (n=134) with no formal education. In the latest Demographic and Health Survey, the proportion of Addis Ababa respondents with higher education was 27.7% only [19]. Although this survey is more than 8 years old, it is possible that our sample is wealthier and more educated than the general population, as they represent health system users, and half of them were enrolled in private health facilities. This may overrepresent the private sector share of health care services delivered in the city. The participants were selected nearly equally from each of the 4 health facility types, including public hospitals (n=628, 26%), health centers (n=624, 25.8%), private clinics (n=621, 25.7%), and private hospitals (n=542, 22.4%).

**Table 3:** Characteristics of the interactive voice response (IVR) sample by participation status among those enrolled.

Variable			Participants^a^ (n=1584), n (%)		Nonparticipants (n=831), n (%)		Total enrolled (n=2415), n (%)
**Sex**							
	Female	908 (57.3)		490 (58.9)		1398 (57.9)	
	Male	676 (42.7)		341 (41)		1017 (42.1)	
**Age group (years)**							
	18-29	623 (39.3)		278 (33.4)		901 (37.3)	
	30-39	516 (32.6)		249 (30)		765 (31.7)	
	40-49	219 (13.8)		145 (17.5)		364 (15.1)	
	≥50	226 (14.3)		159 (19.1)		385 (15.9)	
**Education**							
	No education at all	67 (4.2)		67 (8.1)		134 (5.6)	
	Primary education	326 (20.6)		172 (20.8)		498 (20.6)	
	Secondary education	436 (27.5)		229 (27.6)		665 (27.6)	
	Higher education	755 (47.7)		361 (43.6)		1116 (46.3)	
**Care type**							
	Maternal or reproductive	374 (23.7)		222 (26.8)		596 (24.8)	
	Communicable disease	106 (6.7)		58 (7)		164 (6.8)	
	Noncommunicable disease	661 (41.9)		347 (42)		1008 (41.9)	
	Diarrhea and respiratory	436 (27.7)		200 (24.2)		636 (26.5)	
**Language**							
	Afaan Oromo	274 (17.3)		106 (12.8)		380 (15.7)	
	Amharic	1310 (82.7)		725 (87.2)		2035 (84.3)	
**Preferred survey time**							
	Morning (8-11:59 AM)	319 (20.1)		155 (18.7)		474 (19.6)	
	Mid-day (12-1:59 PM)	323 (20.4)		193 (23.2)		516 (21.4)	
	Afternoon (2-5:59 PM)	269 (17)		173 (20.8)		442 (18.3)	
	Evening (6-7:59 PM)	320 (20.2)		164 (19.7)		484 (20)	
	Night (8-10 PM)	353 (22.3)		146 (17.7)		499 (20.7)	
**Facility type**							
	Health centers	443 (28)		181 (21.8)		624 (25.8)	
	Public hospitals	411 (26)		217 (26.1)		628 (26)	
	Private hospitals	333 (21)		209 (25.2)		542 (22.4)	
	Private clinic	397 (25.1)		224 (27)		621 (25.7)	

^a^This shows the characteristics of those enrolled based on their participation status in the interactive voice response survey (answered at least 1 question or not).

Regarding the type of care received, around 41.9% (n=1008) of the participants sought care for noncommunicable diseases (hypertension, diabetes, or kidney disease), 26.5% (n=637) were treated for diarrhea or respiratory conditions, 24.8% (n=596) received maternal or reproductive health care, and 6.8% (n=164) received care for communicable diseases (including tuberculosis, HIV/AIDS, or sexually transmitted infections). The majority of respondents (n=2035, 84.3%) spoke Amharic. Preferences for survey times were fairly evenly distributed between morning, mid-day, afternoon, evening, and night time.

### Survey Costs and Data Quality

A total of US $13,512 was spent to implement the survey, but 39.3% of these costs (US $5316) were driven by in-person enrollment, which was necessary to obtain the patients’ phone numbers (Table 4). Health facilities, governments, or researchers that can obtain patient phone numbers directly from health records could avoid this portion of the cost. Excluding the in-person expenses, the IVR survey costs were US $7.90 per respondent who completed the full survey (n=1037). Considering those who answered at least 1 question (n=1584), the survey costs were estimated at US $5.17 per partial survey. Of 2415 patients called, only 1584 answered at least 1 question. A total of 831 patients answered no survey questions. The costs for these patients were negligible and included only the cost of the SMS primer and of any airtime used during which the patient may have listened to the introduction and the first question without answering.

**Table 4:** Survey costs.

		Costs (US $)
**In-person enrollment**		
	Including enumerator payments, transport, training, and supplies	5316
**Interactive voice response survey costs**		
	Survey translation and recording	300
	SMS primer (2415 SMS text messages sent)	1017
	Survey airtime	5439
	Incentive (airtime top-up)	1440
Total		13,512

As expected, the number of respondents answering the survey questions declined as the survey progressed, and 34.5% (547/1584) of respondents who began the survey did not complete all questions. For 11 of 15 questions, fewer than 2% of respondents chose to skip the question by pressing “9.” However, there were 3 notable exceptions. First, 36.5% (n=578) of respondents opted to skip the first question, which asked them to rate the cleanliness of the facility. Despite this, most participants went on to answer the second question and continued the survey. This high skip rate is likely because respondents were testing the survey technology on the first question. Future implementers should consider adding a test question at the beginning of their survey, given the high skip rate for our first survey question. Second, for question 13, which informed participants that only 3 questions remained, 16.1% (n=184) chose to skip this question, which asked about the affordability of the services received. Finally, 6.9% (n=72) of respondents skipped the last question, which asked about their monthly household income.

### Factors Affecting Participation in and Completion of the IVR Survey

A Heckman sample selection model was used to predict covariates associated with IVR survey participation (answering at least 1 question) and completion. Before executing this model, we assessed its appropriateness against the standard probit model. The results indicated a correlation between the error terms of the 2 equations, as the value of athrho was statistically significant (*P*<.001). Furthermore, the findings showed a dependence between the selection model and the outcome model, justifying the use of the Heckman probit model since *ρ* is significantly different from 0 (*χ*^2^_1_=9.2; *P*=.002). Additionally, the likelihood function of the Heckman probit model was significant (Wald *χ*^2^_19_=269.9; *P*<.001), indicating the better explanatory power of the Heckman probit model.

The model showed that as the age of the participants increased, the likelihood of both participation and completion declined (Table 5). For example, there was a significant difference in the odds of participating in (odds ratio [OR] 0.77, 95% CI 0.65-0.90) and completing the IVR survey (OR 0.63, 95% CI 0.53-0.74) between participants aged 50 years and older compared to those aged 18-30 years. Similarly, higher education increased the odds of participation and completion. For instance, for respondents with postsecondary education, the odds of participation were 1.54 times higher (95% CI 1.25-1.90) and the odds of survey completion were 1.74 times higher (95% CI 1.46-2.06) compared to those with no education.

**Table 5:** Results of the Heckman probit model.

Variables		Completion of the IVR^a^ survey (2402 observations), OR^b^ (95% CI)	Participation in the IVR survey (2402 observations), OR (95% CI)
**Sex (reference=female)**			
	Male	1.010 (0.907-1.125)	0.983 (0.880-1.097)
**Age category (years) (reference 18-30 years)**			
	30-39	0.853^c^ (0.757-0.960)	0.958 (0.846-1.084)
	40-49	0.666^c^ (0.569-0.779)	0.785^c^ (0.668-0.921)
	≥50	0.628^c^ (0.534-0.739)	0.765^c^ (0.648-0.904)
**Education level (reference=no education)**			
	Primary	0.993 (0.831-1.188)	1.339^c^ (1.081-1.658)
	Secondary	1.318^c^ (1.111-1.563)	1.337^c^ (1.085-1.648)
	Above secondary	1.737^c^ (1.462-2.063)	1.540^c^ (1.248-1.901)
**Time of the survey (reference=morning [8-11:59 AM])**			
	Mid-day (12-1:59 PM)	1.017 (0.873-1.184)	0.861^d^ (0.735-1.007)
	Afternoon (2-5:59 PM)	0.963 (0.819-1.133)	0.826^e^ (0.699-0.975)
	Evening (6-7:59 PM)	0.971 (0.831-1.135)	0.920 (0.783-1.081)
	Night (8-10 PM)	1.163^d^ (0.995-1.361)	1.047 (0.888-1.236)
**Facility type (reference=public health center)**			
	Public hospital	0.866^d^ (0.748-1.002)	0.920 (0.791-1.070)
	Private hospital	0.797^c^ (0.677-0.938)	0.724^c^ (0.611-0.857)
	Private clinic	0.840^e^ (0.727-0.972)	0.819^c^ (0.705-0.952)
**Number of reconnection (reference=0)**			
	One	0.588^c^ (0.514-0.673)	—^f^
	Two or more	0.472^c^ (0.365-0.611)	—
**Type of care (reference=NCD^g^)**			
	Maternal and reproductive	0.804^c^ (0.699-0.925)	0.809^c^ (0.700-0.934)
	Communicable diseases	0.950 (0.775-1.165)	0.904 (0.735-1.110)
	Diarrhea and respiratory	0.972 (0.854-1.107)	0.992 (0.867-1.135)
**Language (reference=Afaan Oromo)**			
	Amharic	—	0.852^c^ (0.761-0.954)
Athrho		— (10,193-1.090e+09)	—
Constant		—	1.810^c^ (1.452-2.256)

^a^IVR: interactive voice response.

^b^OR: odds ratio.

^c^*P*<.01.

^d^*P*<.1.

^e^*P*<.05.

^f^Not applicable.

^g^NCD: noncommunicable disease.

We also found that participation in and completion of the IVR survey was also affected by time of day. Those who were called in the afternoon had lower odds of participating compared to those called in the morning (OR 0.84, 95% CI 0.70-0.98). There was no difference in survey completion according to time of day.

Compared to patients who used services in health centers, those from private hospitals and private clinics were less likely to participate in and complete the survey. For example, primary care patients from private hospitals were substantially less likely to participate in (OR 0.72, 95% CI 0.61-0.86) and complete (OR 0.80, 95% CI 0.68-0.95) the survey compared to those in health centers.

Participants who had received maternal and reproductive health services were less likely to participate in and complete the IVR survey compared to those who received care for noncommunicable diseases. Another important factor identified in determining the completion of the IVR survey was the number of reconnection attempts made. The results revealed that as the number of reconnections increased, participants were less likely to complete the survey. For example, there was a significant difference in completion rates between participants reconnected twice and those not reconnected with an OR of 0.47 (95% CI 0.37-0.61).

### Factors Affecting the Time Taken to Complete the IVR Survey

Results from the Weibull model revealed duration dependency, with a *P* value greater than 1 (*P*=5.037), implying that the hazard function increased over time. This suggests that IVR survey participants were more likely to complete the survey as time progressed. The results indicated that sex, age, time of day, language, and facility type were associated with the duration of IVR survey completion (Table 6).

**Table 6:** Results from Weibull regression (1034 observations).

Variables		Hazard rate (95% CI)
**Sex (reference=female)**		
	Male	1.148^a^ (1.005-1.311)
**Age category (years) (reference<30 years)**		
	30-39	0.957 (0.828-1.106)
	40-49	0.933 (0.759-1.146)
	≥50	0.744^a^ (0.589-0.941)
**Education level (reference=no education)**		
	Primary	0.867 (0.565-1.329)
	Secondary	0.972 (0.641-1.476)
	Above secondary	1.243 (0.824-1.876)
**Time of the IVR^b^ (reference=morning [8-11:59 AM])**		
	Mid-day (12-1:59 PM)	1.139 (0.929-1.396)
	Afternoon (2-5:59 PM)	1.034 (0.834-1.282)
	Evening (6-7:59 PM)	1.078 (0.878-1.323)
	Night (8-10 PM)	1.293^a^ (1.063-1.574)
**Language (reference=Afaan Oromo)**		
	Amharic	1.911^c^ (1.607-2.273)
**Facility type (reference=health center)**		
	Public hospital	1.003 (0.836-1.203)
	Private hospital	1.210^a^ (1.003-1.459)
	Private clinic	1.164 (0.967-1.400)
**Type of care (reference=NCD^d^)**		
	Maternal and reproductive	1.140 (0.950-1.367)
	Communicable diseases	0.983 (0.759-1.272)
	Diarrhea and respiratory	1.154^e^ (0.988-1.347)
*P* value		5.037^c^ (4.826-5.258)
Constant		0^c^ (0-0)

^a^*P*<.05.

^b^IVR: interactive voice response.

^c^*P*<.01.

^d^NCD: noncommunicable disease.

^e^*P*<.10.

Male participants were 1.15 times more likely to complete the survey early compared to female participants, controlling for other factors. As age increased, individuals were less likely to complete the survey early. For instance, the odds of completing the IVR survey quickly for participants aged 50 years and older was 0.74 (95% CI 0.59-0.94) compared to those participants aged 18 to 30 years. The odds of completing the IVR survey faster were higher at night (OR 1.29, 95% CI 1.06-1.57) compared to morning calls. The odds of completing the survey faster were higher for those enrolled in private hospitals (OR 1.21, 95% CI 1.03-1.46) compared to those from health centers. The regression model also controlled for survey language as a proxy for geographical area. Most people speaking Afaan Oromo were from rural areas surrounding Addis Ababa, while those responding in Amharic were from the urban area. The results showed that participants responding in Amharic were more likely to complete the survey early with an OR of 1.91 (95% CI 1.61-2.27).

## Discussion

### Principal findings

This study assessed the feasibility of using an IVR survey as an alternative approach to collect patient satisfaction data in Addis Ababa, Ethiopia. The findings demonstrate a high rate of mobile phone ownership and a willingness among patients to participate, indicating that IVR surveys can serve as a viable low-cost alternative to traditional survey methods to assess patient satisfaction in similar urban areas in a low-income country. Notably, 92.6% (n=2236) of those enrolled picked up the call, 65.6% (n=1584) answered at least 1 survey question, and 42.9% (n=1037) completed the full survey in an average time of 8.1 (SD 1.7) minutes. In adjusted regression models, being older, having less education, and using private facilities negatively affected survey participation and completion. In contrast, being male, being younger, speaking Amharic, using a private hospital, and being called after 8 PM were associated with a shorter survey duration.

The completion rate of 42.9% (n=1037) is encouraging, as almost all of the participants had limited experience with this type of technology. Previous IVR survey studies in Bangladesh, Burkina Faso, Tanzania, and Uganda have found varying response rates ranging from 1% to 41% [20-22]. Response rates were affected by the sampling methods (in-person household surveys or random digit dialing) and type of incentives. Our study, which sampled respondents in person in health facilities and used incentives, achieved a slightly higher response rate (n=1037, 42.9%) than previous studies. In the United States, IVR surveys have been used in various patient satisfaction studies including after endoscopies [11] and for child and adolescent psychiatric outpatient treatment [8]. According to EngageSpark, a global survey and engagement platform, IVR campaigns generally have higher acceptance rates than SMS campaigns. IVR surveys typically achieve response rates ranging from 25% to 75%, while the acceptance rate for SMS surveys ranges from 5% to 15% [23].

Our findings also highlighted the challenges of conducting an IVR survey. For instance, around 16.6% (n=400) of participants listened to the introduction but hung up before answering any questions, and the other 10.4% (n=252) failed to answer the first question despite staying on the call. This suggests that while the initial engagement was successful, there may be factors in the introduction or in the survey’s initial questions that could be optimized to retain participants’ attention or improve their understanding of the system.

We identified several factors influencing participation and completion of the IVR survey. Age emerged as a significant factor, with younger participants being more likely to participate in and complete the IVR survey compared to older participants. This finding aligns with previous studies suggesting that younger individuals are more comfortable with technology and value the privacy that IVR surveys can provide [24]. Education level also significantly impacts both participation and completion rates, with individuals with higher education levels being more likely to engage with IVR surveys. This supports the notion that higher education correlates with better use of technology and higher perceived importance of surveys [25,26].

The type of facility visited by participants influenced the likelihood of participating in and completing the survey. Participants from private hospitals and clinics were less likely to engage with the survey compared to those from health centers. This could be due to the perception that feedback is more likely to improve services in public facilities, which typically serve poorer segments of the population. Another potential reason could be the provision of incentives that attracted lower-income individuals who are more likely to use affordable health care from public health centers [27]. Our incentive consisted of approximately US $0.86 mobile top-up for those who completed the full survey.

The Weibull model for factors associated with the time taken to complete the survey suggested that the hazard function increases over time and that participants were more likely to complete the IVR survey as time progressed [28,29]. The regression results showed that younger individuals tend to complete the survey faster likely due to their greater familiarity with technology. This finding is consistent with literature indicating that older adults may face more challenges with technology, leading to longer completion times [20,30]. The time of day when the IVR survey was conducted also affected completion times. Surveys conducted at night were completed faster compared to those conducted in the morning. This could be due to participants having fewer distractions at night or that those completing the survey at night tend to be more educated. This finding aligns with the idea that identifying optimal times for survey calls can increase participation [24].

Although participants from private hospitals were less likely to participate in and complete the survey, those who completed it were more likely to have a shorter survey duration compared to those from health centers. This may be because higher-income individuals, who are more likely to visit private facilities, are more efficient in completing telephone-based surveys. Language, used as a proxy for area of residence, shows that participants responding in Amharic were more likely to complete the survey faster compared to those responding in Afaan Oromo. This suggests that urban participants, who are more likely to respond in Amharic, complete the survey faster than rural participants. Access to technology and familiarity with IVR systems in urban and rural areas are likely contributing factors.

To further inform our study, a debrief was organized with data collectors who enrolled participants in person and explained the IVR technology. During enrollment, patients revealed concerns with privacy, confusion about the technology, and a preference for in-person interviews. Some individuals were reluctant to share their mobile numbers due to fears of misuse, and face-to-face interactions were sometimes seen as more confidential. Language barriers also hindered participation, as some patients preferred languages that were not supported by our IVR survey. Technical challenges further complicate IVR use, as participants may struggle to navigate prompts without prior experience and with low educational backgrounds and poor literacy. Clear instructions are crucial for engagement. The study showed that older and less-educated individuals were substantially less likely to participate in the phone-based study. This is consistent with evidence that older people and those with no education have limited digital competencies and thereby limited technological abilities [21,22,31].

### Conclusions

IVR systems offer a promising tool for health system research in urban settings of a low-income country. Nonetheless, the implementation of an IVR survey among patients must be carefully planned and tailored to address local challenges. Addressing privacy concerns and building trust are essential. It is also necessary to ensure language inclusivity to improve participation rates and the overall effectiveness of IVR-based public health surveys. Others have found that providing incentives, such as mobile airtime top-ups, can encourage participation and improve response rates, especially for people of lower socioeconomic status [32,33]. Finally, using simple and common language, particularly for medical terms, may ensure better understanding among participants. In considering whether to implement an IVR survey as opposed to an in-person or a computer-assisted telephone interviewing survey with live interviewers, researchers should consider certain tradeoffs. First, we found that the IVR survey was cheap (US $7.90 per full survey); it was implemented quickly and is highly scalable to large samples as long as patient phone numbers can be obtained. However, although we did not find major data quality gaps, IVR may be more prone to data quality issues if users struggle to understand the questions or to answer correctly on their mobile phone keypads. In low-income country settings, in-person surveys may also be affordable if labor costs are low. However, in-person and computer-assisted telephone interviewing surveys take more time to implement and therefore are less scalable, as the number of interviewers and the time taken to conduct surveys must be multiplied when the sample size increases. With IVR, the survey is programmed once and can be answered simultaneously by thousands of respondents simultaneously. In addition, in-person surveys do not ensure privacy and are more prone to social desirability bias than IVR [6].

IVR is much cheaper than other survey methods. For example, health facility surveys such as the Service Provision Assessment surveys (conducted by the Demographic and Health Survey program) that include nationally representative samples of health facilities and patient exit interviews generally cost between US $800 and US $1500 per surveyed health facility [31]. Similarly, the World Bank estimated that a household survey in Sub-Saharan Africa will cost approximately US $300 per household. In addition, IVR surveys tend to be completed much faster. Our entire study was completed in 2 weeks (most of the time was taken for the in-person recruitment), and all data were immediately available through the IVR platform and ready for analysis [32].

There have been few studies in low- and middle-income countries that have tested the use of IVR to survey patients. To our knowledge, ours is the first study to use IVR to collect patient feedback in Ethiopia. IVR offers a low-cost and rapid solution to survey patients and obtain information on perceived quality and satisfaction. In high-income countries, patient satisfaction surveys are often conducted by mail on the web. These survey modes are largely not possible in Ethiopia. In Ethiopia, IVR surveys are likely to function well for younger and more educated respondents in an urban context. Future research should investigate feasibility and response rates in rural areas. Governments and health facilities should consider integrating IVR surveys into routine operations to gather patient satisfaction data and information on patient-perceived quality of care, which can inform quality improvement initiatives and strategies. By leveraging IVR technology, health systems can obtain timely and actionable feedback from patients, ultimately contributing to improved health care quality and better patient outcomes.

## Appendix

Multimedia Appendix 1 Interactive voice response survey instrument on the quality of primary health care in Addis Ababa health facilities.

## Abbreviations

IVR: interactive voice response
OR: odds ratio

## Data Availability

The datasets generated and during this study are available from the corresponding author on reasonable request.
