# Supplementary material for: Using an Interactive Voice Response Survey to Assess Patient Satisfaction in Ethiopia: Development and Feasibility Study
Source: JMIR Form Res. 2025 Feb 13;9:e67452. doi: 10.2196/67452 (PMC11892327; doi:10.2196/67452)
Supplement: Multimedia Appendix 1 [file formative_v9i1e67452_app1.docx]

**Recorded with a female voice in Amharic and Afaan Oromo**

***Recording 1: Welcome message***

Hello, we are calling from Addis Ababa University to complete a survey on primary care quality. On this call, we will be asking you some questions and you will answer by pressing numbers on your phone. It will take less than 10 minutes to complete. If you complete the entire survey, 50 Ethiopian Birr of airtime will be sent to your phone.

***Recording 2: Q1 SQ_cleanliness***

We would like to ask your opinion about specific aspects of the health care services you received at the facility where you were enrolled in this study.

Thinking about that visit, how would you rate the cleanliness of the health facility environment, including the washrooms?

*To answer the question, use the keypad on your phone.*

*Press the number 1 button on your phone if it was* ***very poor***

*Press the number 2 if it was* ***poor***

*Press the number 3 if it was* ***neither good or poor***

*Press the number 4 if it was* ***good***

*Press the number 5 if it was* ***very good***

To repeat the question press #, to skip the question, press the number 9 on your phone.

***Recording 3: Invalid key press message***

This is not a valid response. Please try again.

***Recording 4: Q2 SQ_equipment***

How would you rate the availability and quality of the medical equipment?

*To answer the question, use the keypad on your phone.*

*Press the number 1 button on your phone if it was* ***very poor***

*Press the number 2 if it was* ***poor***

*Press the number 3 if it was* ***neither good or poor***

*Press the number 4 if it was* ***good***

*Press the number 5 if it was* ***very good***

To repeat the question press #.

***Recording 5: Q3 SQ_diagnotics***

How would you rate the availability of diagnostic tests such as blood tests, urine tests or imaging?

*To answer the question, use the keypad on your phone.*

*Press the number 1 button on your phone if it was* ***very poor***

*Press the number 2 if it was* ***poor***

*Press the number 3 if it was* ***neither good or poor***

*Press the number 4 if it was* ***good***

*Press the number 5 if it was* ***very good***

To repeat the question press #.

***Recording 6: Q4 SQ_medicine***

How would you rate the availability of medicine, including pills, vaccines, injections, or other treatments?

*To answer the question, use the keypad on your phone.*

*Press the number 1 button on your phone if it was* ***very poor***

*Press the number 2 if it was* ***poor***

*Press the number 3 if it was* ***neither good or poor***

*Press the number 4 if it was* ***good***

*Press the number 5 if it was* ***very good***

To repeat the question press #.

***Recording 7: Q5 CC_skills***

Regarding the health providers and staff, how would you rate the competence of the health care providers including their knowledge and skills?

*To answer the question, use the keypad on your phone.*

*Press the number 1 button on your phone if it was* ***very poor***

*Press the number 2 if it was* ***poor***

*Press the number 3 if it was* ***neither good or poor***

*Press the number 4 if it was* ***good***

*Press the number 5 if it was* ***very good***

To repeat the question press #.

***Recording 8: Q6 CC_explanations***

How would you rate the clarity of the explanations or advice received from the healthcare providers at this facility?

*To answer the question, use the keypad on your phone.*

*Press the number 1 button on your phone if it was* ***very poor***

*Press the number 2 if it was* ***poor***

*Press the number 3 if it was* ***neither good or poor***

*Press the number 4 if it was* ***good***

*Press the number 5 if it was* ***very good***

To repeat the question press #.

***Recording 9: Q7 CC_solve***

How would you rate the ability of the providers to solve most of your health problems or health concerns?

*To answer the question, use the keypad on your phone.*

*Press the number 1 button on your phone if it was* ***very poor***

*Press the number 2 if it was* ***poor***

*Press the number 3 if it was* ***neither good or poor***

*Press the number 4 if it was* ***good***

*Press the number 5 if it was* ***very good***

To repeat the question press #.

***Recording 10: Q8 CC_coordination***

How would you rate the helpfulness of providers or staff in coordinating your care. This could include helping you get appointments, following up or helping you receive care from other facilities or providers.

*To answer the question, use the keypad on your phone.*

*Press the number 1 button on your phone if it was* ***very poor***

*Press the number 2 if it was* ***poor***

*Press the number 3 if it was* ***neither good or poor***

*Press the number 4 if it was* ***good***

*Press the number 5 if it was* ***very good***

To repeat the question press #.

***Recording 11: Q9 RC_respect***

Regarding respect and customer service, how would you rate the level of respect you received from health care providers or staff at this facility?

*To answer the question, use the keypad on your phone.*

*Press the number 1 button on your phone if it was* ***very poor***

*Press the number 2 if it was* ***poor***

*Press the number 3 if it was* ***neither good or poor***

*Press the number 4 if it was* ***good***

*Press the number 5 if it was* ***very good***

To repeat the question press #.

***Recording 12: Q10 RC_privacy***

How would you rate the level of privacy you had during your consultations. This means whether other clients or people could see or hear you.

*To answer the question, use the keypad on your phone.*

*Press the number 1 button on your phone if it was* ***very poor***

*Press the number 2 if it was* ***poor***

*Press the number 3 if it was* ***neither good or poor***

*Press the number 4 if it was* ***good***

*Press the number 5 if it was* ***very good***

To repeat the question press #.

***Recording 13: Q11 PC_hours***

How would you rate the convenience of the facility’s opening hours?

*To answer the question, use the keypad on your phone.*

*Press the number 1 button on your phone if it was* ***very inconvenient***

*Press the number 2 if it was* ***inconvenient***

*Press the number 3 if it was* ***neither convenient or inconvenient***

*Press the number 4 if it was* ***convenient***

*Press the number 5 if it was* ***very convenient***

To repeat the question press #.

***Recording 14: Q12 PC_wait***

How would you rate the amount of time you waited before seeing a provider at this facility?

*To answer the question, use the keypad on your phone.*

*Press the number 1 button on your phone if it was* ***very long***

*Press the number 2 if it was* ***long***

*Press the number 3 if it was* ***neither short or long***

*Press the number 4 if it was* ***short***

*Press the number 5 if it was* ***very short***

To repeat the question press #.

***Recording 15: Q13 Cost***

We have only three more questions.

How affordable were the health care services your received from the facility?

Consider any money spent on the consultation, medicines, vaccines, or laboratory tests.

*To answer the question, use the keypad on your phone.*

Press 0 if the health care services were free.

Press 1 if the health care was very affordable.

Press 2 if the health care was affordable.

Press 3 if the health care was expensive.

Press 4 if the health care was very expensive

*To repeat the question press #.*

***Recording 16: Q14 Recommend***

How likely is it that you would recommend this health facility to a friend or family member?

*Press the number 1 button on your phone if you are* ***not at all likely to recommend the facility***

*Press the number 2 if you are* ***not likely to recommend the facility***

*Press the number 3 if it* ***likely to recommend the facility***

*Press the number 4 if it* ***you are*** ***very likely to recommend the facility***

To repeat the question press #.

***Recording 17: Q15 income***

This is the last question.

If you think about your total monthly household income, which of these categories does it fit into?

Press 1 if your monthly household income is Less than 1000 Birr.

Press 2 if your monthly household income is between 1000 and 3000 Birr

Press 3 if your monthly household income is between 3001 and 5000 Birr

Press 4 if your monthly household income is between 5001 and 10000 Birr

Press 5 if your monthly household income is between 10001 and 20000 Birr

Press 6 if your monthly household income is Greater than 20000 Birr

To repeat the question press #.

***Recording 18: Closing message***

Thank you very much for taking the time to answer this survey. Airtime will be credited to your mobile phone. Goodbye

***Recording 19: Failure message***

Unfortunately, we did not receive a valid answer. We will now end the survey. Goodbye.
